# Impaired kidney function among young healthcare workers with long working hours and night work

**DOI:** 10.5271/sjweh.4159

**Published:** 2024-07-01

**Authors:** Wan-Chin Chen, Hsiao-Yu Yang MD

**Affiliations:** 1 Department of Family Medicine, Changhua Christian Hospital, Changhua City, Taiwan.; 2 Institute of Environmental and Occupational Health Sciences, National Taiwan University College of Public Health, No. 17 Xuzhou Road, Zhongzheng Dist., Taipei, Taiwan; 3 Department of Environmental and Occupational Medicine, National Taiwan University Hospital, No. 1, Changde St., Zhongzheng Dist., Taipei, Taiwan.; 4 Population Health Research Center, National Taiwan University, Taipei, Taiwan.

**Keywords:** early renal dysfunction, glomerular filtration rate

## Abstract

**Objectives:**

We aimed to evaluate the association between long working hours, night work, and estimated glomerular filtration rate (eGFR) among young healthcare workers.

**Methods:**

We conducted a retrospective cohort study among healthcare workers in a tertiary medical center in Taiwan from 2002 to 2021. Other than physicians, all hospital employees aged 20–65 years with documented yearly working hours and an annual blood test including creatinine were eligible. We excluded participants with eGFR <60 ml/min/1.73 m^2^ and proteinuria at enrollment to focus on early renal impairment. Total working hours, night working hours, and eGFR in each year were collected. We assessed the relationship of total working hours and night and non-night working hours with eGFR using the generalized linear mixed model, adjusting for demographic, comorbidities, and laboratory profiles.

**Results:**

The study included 10 677 participants with a mean age of 27.2 (standard deviation 7.1) years. The mean follow-up duration was 6.2 years. For every 10-hour increase in total weekly working hours, the eGFR decreased by 0.86 [95% confidence interval (CI) 0.61–1.11] ml/min/1.73 m^2^. For every 10-hour increase in weekly night working hours, the eGFR decreased by 0.25 (95% CI 0.07–0.42) ml/min/1.73 m^2^. In stratified analysis, the negative associations between total working hours and eGFR remained in the subgroups of individuals aged <40 years and those without hypertension or diabetes, with a P-value for interaction of <0.05.

**Conclusions:**

Longer working hours and night work were associated with lower eGFR among healthcare workers.

Chronic kidney disease (CKD) has increased rapidly worldwide with a global prevalence of 9.1% (1). Recognized as the 12^th^ leading cause of death globally, CKD accounts for 1.2 million deaths annually (2). In Taiwan, the prevalence of CKD is estimated at 15.46% overall and 9.06% for stages 3 to 5 (3). Taiwan has the highest incidence (0.455/1,000 person-years) and prevalence (0.32%) of end-stage renal disease among international countries surveyed (4). While diabetes and hypertension are the primary causes of CKD (5), exploring other risk factors is important for a comprehensive understanding and management of kidney disease. Beyond these traditional risk factors, it is imperative to identify additional contributors to early renal dysfunction to mitigate the CKD burden.

Long working hours are not only a significant occupational concern but also a public health issue. According to the Ministry of Labor, in 2022, average annual working hours in Taiwan reached 2008, higher than the Organization for Economic Cooperation and Development's numbers for South Korea (1901 hours) and Japan (1607 hours). This places Taiwan among the top ten countries globally for high working hours in recent years. Long working hours are associated with a variety of adverse health outcomes, including physical health problems like sleep disorders and coronary heart disease, as well as mental disorders such as depression, anxiety, and higher suicide mortality rates (6, 7). Previous cross-sectional studies have indicated an association between long working hours and renal impairment (8, 9). Additionally, there has been only one cohort study that has demonstrated a relationship between long working hours and the incidence of CKD (10). Thus, the association between long working hours and CKD needs more attention.

The prevalence of shift work has surged globally in line with economic development. Shift work includes various work schedules occurring outside of typical daytime working hours, such as night shift or night work. In the United States, over 27 million workers are engaged in flexible work schedules, with 14.8% predominantly working shifts other than regular daytime hours (11). The proportion of shift work is high in Asian countries, in particular in Taiwan (25%) (12). Shift work, especially night shifts, is associated with decreased kidney function (13). In addition, long duration of night shift work years is associated with early stage of renal dysfunction (14).

A major limitation among the studies about long working hours and CKD is inconsistent assessment of long working hours exposure. Long working hours were evaluated using a self-report questionnaire, and a shorter follow-up period to develop CKD was also a limitation. The same restrictions in the literature of night shift work and CKD were also noted. Although some epidemiological studies reported a positive association between night shift work and CKD, the assessment of night shift work exposure was acquired by self-report questionnaires, which might suffer from recall bias owing to complex rotating work schedules. Studies that can provide a more accurate quantitative measure of total working hours and night working hours are still lacking.

We hypothesize that long working hours and night work increase the risk of renal dysfunction, reflected by lower estimated glomerular filtration rate (eGFR) in the early stage. Our study primarily aims to establish the association of long working hours and night work with early renal impairment, rather than overt CKD, among young healthcare workers (HCW). Particularly, we try to identify subgroups at risk of early renal impairment related to working hours.

## Methods

### Study design and participants

We conducted a retrospective cohort study at a medical center in central Taiwan, which has approximately 1450 beds. The data collection spanned from January 2002 to December 2021.

**Figure 1:**
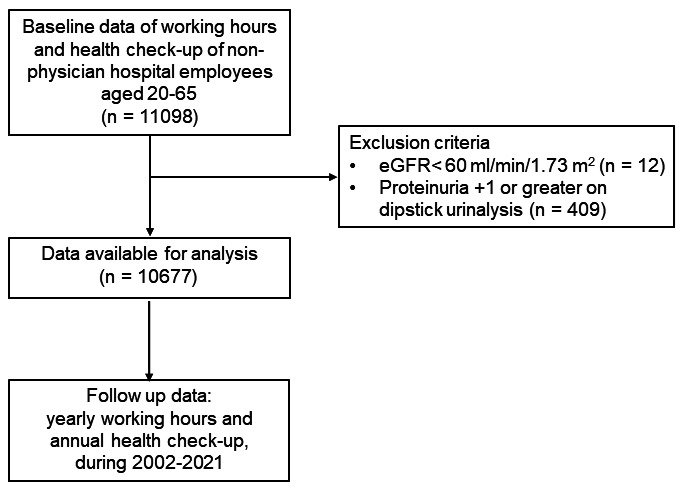
Flowchart for the selection of eligible participants.

Participants eligible for the study included all hospital employees aged 20–65 years, excluding physicians, who worked at the hospital during this period and had documented actual working hours as well as annual health examinations (N=11 098). The annual health exam, regulated by the employer, should be completed by the end of the year. We excluded participants with suspected CKD (eGFR <60 ml/min/1.73 m^2^) within the year of entry (N=12). We also excluded those with proteinuria +1 or greater on dipstick urinalysis (N=409). Ultimately, our analysis included 10 677 participants, comprising 9482 women and 1195 men (figure 1).

### Ascertainment of night work and total working hours

In our study, we defined night working hours as 0:00–08:00 hours. Total working hours were considered as the sum of all working hours, encompassing both non-night and night work. Consequently, non-night working hours were calculated by subtracting night working hours from the total. We systematically collected data on both non-night and night working hours of participants every year. Furthermore, work patterns were divided into two categories: (i) non-shift work: a worker engaged only in day work and (ii) shift work: a worker not limited to day work alone.

In Taiwan, the Labor Standards Act governs the working hours of hospital employees, with the exception of physicians whose working hours are not regulated. Thus they were not enrolled into this study. Hospitals are mandated to report the working hours of HCW to the Department of Labor each month. These hours are directly tied to the employees’ salaries and are accurately recorded. To eliminate recall bias, we accessed these working hours through an electronic recording system.

### Demographics, blood tests and anthropometric data

Demographic data, including age and sex were obtained from electronic medical records. Personal habits such as smoking, drinking, and exercise were assessed by questionnaires completed during employees' annual health check-up. Anthropometric data, including body weight, height, and blood pressure, were retrieved from medical records. Blood pressure was measured twice using an electronic device. Height and weight were measured by a nurse or assistant, with participants in the standing position without shoes or outer garments. Height was measured to the nearest 0.1 cm, and weight was measured to the nearest 0.1 kg with a digital stadiometer. Body mass index (BMI) was calculated as weight (kg) divided by the square of height (m^2^).

During the health check-up, blood was drawn from the antecubital vein after eight hours of overnight fasting. Biochemical data, including total cholesterol, triglycerides, low-density lipoprotein cholesterol (LDL-C), and high-density lipoprotein cholesterol (HDL-C), uric acid, creatinine, glucose, and hemoglobin A1c (HbA1c) were analyzed. Urine protein was checked by urine dipstick analysis from morning spot urine sample. Serum creatinine was measured using the Jaffe rate method (Beckman Coulter’s AU5800 series chemistry analyzers). Estimated GFR was calculated by the 2021 Chronic Kidney Disease Epidemiology Collaboration (CKD-EPI) equation (15). The 2021 CKD-EPI equation is as follows: eGFR = 142 × min (Scr /κ, 1) ^α^ × max (Scr /κ, 1)^-1.200^ × 0.9938^Age^ × (1.012 if female). Scr is serum creatinine in mg/dL, κ is 0.7 for females and 0.9 for males, α is -0.241 for females and -0.302 for males, min indicates the minimum of Scr /κ or 1, max indicates the maximum of Scr /κ or 1.

The College of American Pathologists accredits the laboratory for high-quality assurance and reliability.

### Determination of diagnosed diabetes and hypertension

Because hypertension and diabetes are major risk factors for renal function impairment, we included these two diseases in our analysis. The determination of diagnosed hypertension and diabetes was conducted through the following methods. We obtained the results of annual health checkups and medical records from the hospital’s electronic medical record system. The diagnosis of hypertension was determined by either: (i) a systolic blood pressure ≥140 mmHg or a diastolic blood pressure ≥90 mmHg during annual health check-ups, or (ii) a diagnosis of hypertension by a physician, as indicated by ICD-9 codes 401, 402, 404, 405, or ICD-10 codes I10, I11, I12, I13, I15. Similarly, the diagnosis of diabetes was established by either: (i) a fasting plasma glucose level of ≥126 mg/dL (7.0 mmol/L) or a HbA1c level of ≥6.5% (48 mmol/mol) during annual health check-ups, or (ii) a diagnosis of diabetes by a physician, as indicated by ICD-9 codes 250, 251, or ICD-10 codes E10, E11, E13, during the study period.

### Statistical analysis

We first provided the baseline characteristics of all participants. They were compared across three levels of working hours: <30, 30–40, and >40 hours per week. The Kruskal–Wallis test was used to calculate P-values for continuous variables and the chi-squared test or Fisher’s exact test for categorical variables. The generalized linear mixed model (GLMM) was utilized to evaluate the association of total working hours, night working hours, and non-night working hours with eGFR obtained from the health checkup data in the same year. The GLMM incorporated a random intercept for repeated measurements of each worker. Multivariable adjusted β slope and 95% confidence intervals (CI) were reported, adjusting for covariates of age, sex, hypertension status, diabetes status, BMI, lipid profiles, uric acid, smoking, and drinking. We additionally adjusted for shift work status in the model for total working hours. Furthermore, we performed stratified analyses by age groups (<40 versus ≥40 years old), sex, hypertension status, diabetes status, and uric acid (<6 versus ≥6 mg/dL).

All statistical tests were two-sided, with a significance level of 0.05. The analyses were performed using SAS, version 9.4 (SAS Institute, Cary, NC, USA).

## Results

### Total working hours, night working hours and eGFR

From 2002–2021, our study recruited 10 677 participants, with a mean follow-up duration of 6.2 years. At baseline, the mean age of the participants was 27.2 [standard deviation (SD) 7.1] years, and the mean eGFR was 113.1 (SD 16.5) ml/min/1.73m^2^. A majority of the participants, 88.8%, were female. There were 5969 (55.9%) nurses, 1928 (18.1%) other professionals including technicians, radiologists, therapists, pharmacists, etc., and 2776 (26.0%) other hospital staff. Participants, on average, worked 37.1 hours per week and engaged in night work for 2.9 hours per week. Table 1 shows the baseline characteristics of all participants at the time of enrollment. HCW with higher working hours exhibited lower eGFR. Females consistently made up a larger proportion across different levels of working hours, yet the proportion of females decreased among those who worked >40 hours per week. A higher prevalence of hypertension and a lower prevalence of diabetes were observed among those with longer working hours, although these differences were not statistically significant. The proportion of shift workers, along with nurses, was higher among those who worked 30–40 hours per week. In contrast, the proportion of other professionals increased among those who worked >40 hours per week. Notably, the number of HCW with smoking or drinking habits was rare. The proportion of missing data on exercise was extremely high, leading to its exclusion from further analysis.

**Table 1:** Baseline characteristics among all participants (N=10 677). [SBP=systolic blood pressure; DBP=diastolic blood pressure; BMI=body mass index; TC=total cholesterol; HDL-C=high-density lipoprotein cholesterol; LDL-C=low-density lipoprotein cholesterol; TG=triglycerides; eGFR=estimated glomerular filtration rate; SD=standard deviation]. **Numbers in bold** indicate statistically significant findings (P<0.05).

Variable		Missing		Total (N=10 677)			Total working hours (per week)								P-value ^a^
							<30 (N=753)			30–40 (N=7391)			>40 (N=2533)		
		N		Mean (SD)	N (%)		Mean (SD)	N (%)		Mean (SD)	N (%)		Mean (SD)	N (%)	
Age (years)		0		27.2 (7.1)			26.8 (6.4)			27.4 (7.4)			26.6 (6.5)		**0.005**
BMI (kg/m^2^)		12		22.1 (4.0)			22.4 (4.3)			22.1 (3.9)			21.9 (4.0)		**0.026**
TC (mg/dL)		3		178.5 (32.2)			179.7 (33.9)			177.9 (32.1)			179.9 (31.8)		**0.006**
HDL-C (mg/dL)		6071		58.9 (13.3)			57.7 (14.0)			59.0 (13.2)			58.8 (13.6)		0.141
LDL-C (mg/dL)		6075		104.5 (28.9)			104.5 (29.6)			103.8 (28.5)			108.0 (30.1)		**0.010**
TG (mg/dL)		5		75.9 (51.9)			82.1 (77.7)			76.0 (50.6)			73.8 (45.7)		0.052
Uric acid (mg/dL)		3467		5.1 (1.2)			5.1 (1.2)			5.1 (1.2)			5.2 (1.3)		**0.006**
Creatinine (mg/dL)		0		0.7 (0.2)			0.7 (0.2)			0.7 (0.2)			0.8 (0.2)		**<0.001**
eGFR (ml/in/1.73m^2^)		0		113.1 (16.5)			116.0 (15.2)			113.2 (16.8)			111.9 (15.8)		**<0.001**
Total working hours (per week)		0		37.1 (5.6)			20.8 (7.0)			37.2 (2.1)			41.7 (1.9)		**<0.001**
Night working hours (per week)		0		2.9 (5.0)			0.9 (2.5)			3.3 (5.3)			2.4 (4.1)		**<0.001**
Female sex		0			9482 (88.8)			673 (89.4)			6683 (90.4)			2126 (83.9)	**<0.001**
Hypertension		0			862 (8.1)			57 (7.6)			573 (7.8)			232 (9.2)	0.071
Diabetes		0			134 (1.3)			11 (1.5)			93 (1.3)			30 (1.2)	0.835
Current smoker		8604			18 (0.9)			1 (0.6)			11 (0.7)			6 (1.8)	0.160 *
Alcohol drinking		8635			17 (0.8)			3 (1.8)			12 (0.8)			2 (0.6)	0.305 *
Shift work		0			6206 (58.1)			295 (39.2)			4653 (63.0)			1258 (49.7)	**<0.001**
Occupation		4													**<0.001**
	Nurse				5969 (55.9)			404 (53.7)			4579 (62.0)			986 (39.0)	
	Other professionals ^b^				1928 (18.1)			109 (14.5)			1091 (14.8)			728 (28.8)	
	Other hospital staff				2776 (26.0)			239 (31.8)			1720 (23.3)			817 (32.3)	

^a^ P-values were calculated by Kruskal–Wallis test for continuous variables, and the chi-squared test or Fisher’s exact test (*) for categorical variables. Numbers in bold indicate statistically significant findings (P<0.05). ^b^ Other professionals include technicians, radiologists, therapists, pharmacists, etc.

Table 2 presents the associations of total, night, and non-night weekly working hours with eGFR as determined by the GLMM. We adjusted for several variables, including age, sex, hypertension, diabetes, BMI, lipid profiles, smoking and drinking, and additionally, shift work status in Model 1. In terms of eGFR outcomes, we observed that for every 10-hour increase in total weekly working hours, the eGFR decreased by 0.86 ml/min/1.73m^2^ (95% CI 0.61–1.11). The effect size was comparable to the effect of aging, in that the eGFR was 0.76 ml/min/1.73m^2^ lower for every year older at baseline age and declined by 0.88 ml/min/1.73m^2^ for every 1-year increase in age. Similarly, for every 10-hour increase in weekly night working hours, there was a decrease of 0.25 ml/min/1.73m^2^ in eGFR (95% CI 0.07–0.42). However, we found no statistically significant association between higher non-night working hours and lower eGFR. In all models, several factors were consistently associated with lower eGFR: older age at baseline, longer follow-up time, male sex, hypertension, higher LDL-C, and higher uric acid levels. In the model analyzing total working hours, individuals with shift work exhibited lower eGFR compared to those without shift work.

**Table 2:** Fixed effects parameters of the generalized linear mixed model with estimated glomerular filtration rate (2021 CKD-EPI) as outcome (N=10 677). [SBP=systolic blood pressure; DBP=diastolic blood pressure; BMI=body mass index; HDL-C=high-density lipoprotein cholesterol; LDL-C=low-density lipoprotein; TG=triglycerides; eGFR=estimated Glomerular filtration rate; CI=confidence interval]. **Numbers in bold** indicate statistically significant findings (P<0.05).

		Model 1 ^a^		Model 2 ^a^		Model 3 ^a^
		β (95% CI)		β (95% CI)		β (95% CI)
Working hours						
	Total ^b^	**-0.86 (-1.11– -0.61)**				
	Night ^b, c^			**-0.25 (-0.42 – -0.07)**		
	Non-night ^b^					-0.11 (-0.27–0.04)
Age (years)		**-0.76 (-0.79– -0.73)**		**-0.76 (-0.79– -0.73)**		**-0.75 (-0.78– -0.72)**
Follow-up time (years)		**-0.88 (-0.91– -0.84)**		**-0.87 (-0.90– -0.83)**		**-0.86 (-0.90– -0.83)**
Male sex		**-4.42 (-5.39– -3.44)**		**-4.47 (-5.45– -3.50)**		**-4.42 (-5.40– -3.45)**
Hypertension		**-0.37 (-0.70– -0.04)**		**-0.39 (-0.72– -0.07)**		**-0.41 (-0.74– -0.09)**
Diabetes		-0.09 (-0.90–0.73)		-0.08 (-0.90–0.74)		-0.08 (-0.90–0.74)
BMI (kg/m^2^)		**0.33 (0.26–0.40)**		**0.35 (0.28–0.42)**		**0.35 (0.28–0.41)**
HDL-C (mg/dL) ^b^		**0.27 (0.16–0.37)**		**0.30 (0.20–0.41)**		**0.30 (0.19–0.41)**
LDL-C (mg/dL) ^b^		**-0.09 (-0.14– -0.03)**		**-0.08 (-0.13– -0.02)**		**-0.08 (-0.14– -0.02)**
TG (mg/dL) ^d^		**0.09 (0.06–0.11)**		**0.10 (0.07–0.12)**		**0.10 (0.07–0.12)**
Uric acid (mg/dL)		**-2.86 (-3.07– -2.66)**		**-2.87 (-3.07– -2.67)**		**-2.87 (-3.07 – -2.67)**
Current smoker		-0.98 (-3.03–1.07)		-0.95 (-3.03–1.12)		-1.00 (-3.07–1.07)
Drinking		-0.52 (-1.50–0.46)		-0.56 (-1.54–0.43)		-0.55 (-1.53–0.43)
Shift work		**-0.29 (-0.56– -0.02)**				

^a^ Model 1, 2, and 3 adopt total working hours, night working hours, and non-night working hours as main predictors, respectively. Model 1 additionally adjusted for shift work status. ^b^ For every 10 hours per week. ^c^ Night working hours were from 0:00–08:00 hours. Total working hours were considered as the sum of all working hours, encompassing both non-night and night working hours. ^d^ For every 10 mg/dL.

### Stratified analysis

Table 3 illustrates the stratified analysis. The aforementioned association between total working hours and eGFR persisted in the subgroups of individuals <40 years, females, those without hypertension, those without diabetes, and irrespective of uric acid levels. The interactions of total working hours with age, hypertension status, and diabetes status were statistically significant (P for interaction <0.05). Similarly, the association between night working hours and eGFR persisted in the subgroups of females, those without hypertension, those without diabetes, those with uric acid levels <6 mg/dL, and irrespective of age.

**Table 3:** Stratified analysis based on age, sex, hypertension, diabetes, and uric acid presented with adjusted 95% confidence intervals (CI) for the relationship between working hours per week and estimated glomerular filtration rate. **Numbers in bold** indicate statistically significant findings (P<0.05).

Variable		N	Total working hours per week ^a^	P for interaction	Night working hours per week ^a^	P for interaction
Age (years)				**0.002**		0.284
	<40	9844	**-1.01 (-1.26– -0.75)**		**-0.26 (-0.43– -0.08)**	
	≥40	833	0.50 (-0.37–1.37)		**-0.66 (-1.32– -0.002)**	
Sex				0.091		0.637
	Female	9482	**-0.90 (-1.15– -0.65)**		**-0.24 (-0.42– -0.07)**	
	Male	1195	-0.17 (-1.15–0.82)		0.02 (-0.91–0.95)	
Hypertension				**0.025**		0.217
	Non-hypertension	9815	**-0.96 (-1.22– -0.71)**		**-0.28 (-0.46– -0.09)**	
	Hypertension	862	0.12 (-0.83–1.08)		0.05 (-0.50–0.60)	
Diabetes				**<0.001**		0.248
	Non-diabetes	10543	**-0.91 (-1.16– -0.67)**		**-0.25 (-0.42– -0.07)**	
	Diabetes	134	1.76 (-0.17–3.69)		0.74 (-0.23–1.72)	
Uric acid (mg/dL)				0.559		0.250
	<6	5722	**-0.84 (-1.1 – -0.57)**		**-0.30 (-0.50– -0.11)**	
	≥6	1488	**-0.78 (-1.44– -0.12)**		-0.06 (-0.47**–**0.36)	

^a^ For every 10 hours increase per week.

### Sensitivity analysis

Due to substantial missing data of smoking and drinking habits, we conducted a sensitivity analysis, excluding these two variables from the model. For every 10-hour increase in total weekly working hours, eGFR decreased by 1.25 ml/min/1.73m^2^ (95% CI 1.03–1.47). Similarly, for every 10-hour increase in weekly night working hours, there was a decrease of 0.58 ml/min/1.73m^2^ in eGFR (95% CI 0.41–0.76). These results indicated that the primary findings of our study remained unchanged, with a slightly increased effect size after the removal of information on smoking and drinking habits.

In the supplementary material (www.sjweh.fi/article/4159) table S1, we replaced eGFR from the 2021 CKD-EPI equation with that from the Modification of Diet in Renal Disease (MDRD) equation. For every 10-hour increase in total weekly working hours, eGFR decreased by 3.69 ml/min/1.73m^2^ (95% CI 2.87–4.51). Similarly, for every 10-hour increase in weekly night working hours, there was a decrease of 1.00 ml/min/1.73m^2^ in eGFR (95% CI 0.52–1.48). The effect sizes were larger, but the direction of the associations remained the same. Additionally, non-night working hours were also associated with eGFR, showing borderline statistical significance. We further explored the relationship between eGFR derived from the 2021 CKD-EPI and MDRD equations, and found a strong correlation between the two (Pearson’s correlation coefficient = 0.85, P<0.001). A scatterplot in supplementary figure S1 illustrates that the eGFR derived from the 2021 CKD-EPI and MDRD equations correlate more closely within the low to normal range of eGFR. However, the values derived from MDRD show much larger variation and tend to be inflated once the eGFR exceeds 90 ml/min/1.73m^2^.

In supplementary table S2, we further divided total working hours into three levels (<30, 30–40, and >40 hours per week) and re-evaluated its association with both eGFR derived from the 2021 CKD-EPI and MDRD equations. Compared with the reference group working <30 hours per week, the eGFR (2021 CKD-EPI) decreased by 1.27 (95% CI 0.87–1.66) and 1.23 (95% CI 0.74–1.72) ml/min/1.73m^2^ for every 10-hour increase in weekly working hours among those working 30–40 and >40 hours per week, respectively. The results, when using eGFR derived from the MDRD equation, showed similar patterns but with larger effect sizes.

We also demonstrated a decrease in eGFR (2021 CKD-EPI) of 3.09 (-2.08–8.27) for every 10-hour increase in weekly working hours among those working >40 hours per week (data not shown). However, the trend suggesting that increased working hours are associated with renal impairment did not achieve statistical significance. This lack of significance may be due to the limited statistical power, resulting from the substantial drop in participant numbers as working hours increased further.

## Discussion

This study has demonstrated a clear association on longer working hours and night working hours with lower eGFR, particularly focusing on early renal function impairment in young workers. To the best of our limited knowledge, this is the first study to utilize more accurate working hour measurements in a predominantly young workforce for assessing the impact of working hours on renal function.

The primary strength of our study is the use of more precise data on total and night working hours. We annually gather working hours directly from electronic records to minimize recall bias, along with yearly health examination data that includes creatinine levels. Our participants, mainly comprising young individuals without established CKD, uniquely positions our study to more effectively explore the impact of working hours on renal health. Distinguishing our approach from prior studies, we relied on actual working hours rather than self-reported questionnaires. Furthermore, our study benefits from a comprehensive and representative sample drawn exclusively from a single large medical center. This approach ensures consistency in data collection, analysis equipment, and allows us to access complete profiles of working hours and annual examinations for all employees, significantly reducing the potential for information bias.

Our results confirm the association of longer working hours with lower eGFR, consistent with the outcomes of previous studies. A cross-sectional study of 20 851 predominantly middle-aged workers reported a non-linear decrease in eGFR, noting an attenuated effect for each additional hour beyond 40 hours per week (16). A longitudinal study involving 97 856 healthy workers, with a mean age of 36.4 years, found an elevated risk of CKD among those who worked >52 hours per week, compared with the reference group working 35–40 hours (10). Notably, our study population, was relatively younger, as HCW had greater medical knowledge compared to individuals in other populations. When compared to the reference group working <30 hours per week, those working 30–40 and >40 hours per week exhibited decreases in eGFR of 1.27 and 1.23 ml/min/1.73m^2^, respectively (supplementary table S2). We categorized our participants into 10-hour intervals. However, the small number of individuals working >50 hours per week at baseline (9 females and 1 male) precluded further subdivision among those working >40 hours per week. A previous study recommended gender-specific thresholds for work hours and health impact (43.5 hours for men and 38 hours for women), suggesting that men may have a higher tolerance to long working hours than women (17). In our study, despite the predominance of females, the proportion of men increased in the group working >40 hours per week. This increase may account for the observed lack of a more profound decrement in eGFR after weekly working hours >40 hours.

The underlying mechanisms driving the association between longer working hours and impaired kidney function are not fully understood. Prolonged working hours are often correlated with unhealthy behaviors, such as smoking, alcohol consumption, sedentary lifestyle, and sleep disturbance (18). It is shown that short sleep time and poor sleep quality may have a direct effect on CKD through the renin-angiotensin-aldosterone system and sympathetic nervous system activation (19–21). Additionally, low physical activity and prolonged sedentary time may increase the risk of CKD by increasing insulin and vascular resistance (22–24). Moreover, longer working hours are strongly linked with increased psychosocial stress among employees (25), and higher levels of psychosocial distress with a faster decline in kidney function (26). Stress, known as a risk factor of CKD, may exert its effect through the activation of the hypothalamic‒pituitary‒adrenal axis, inflammatory cytokines, and endothelin-A, leading to sodium and water retention and the progression of CKD (27).

Our research is the first cohort study to establish a link between night working hours and impaired renal function. This finding is consistent with previous research. A cross-sectional study of 354 police officers, which found that a higher percentage of night shift hours correlated with lower eGFR (13). Another cross-sectional study of 6869 steelworkers indicated that long-term night shift work was associated with early-stage renal dysfunction in male workers (14). A plausible mechanism for the observed correlation between longer night working hours and lower eGFR lies in circadian disruption. Nighttime light exposure may disrupt the natural light-dark cycle (28). The circadian system consists of a central brain clock in the hypothalamic suprachiasmatic nucleus and peripheral clocks in tissues such as the kidney. Circadian rhythms are crucial in regulating physiological functions such as blood pressure and renal excretion (29). Working during night hours leads to a disruption of these rhythms, potentially accelerating kidney function decline and increasing the susceptibility to kidney disease.

In our stratified analysis, the association of total and night working hours with renal function was consistently observed in relatively healthy subgroups, characterized by younger age, female sex, those without hypertension or diabetes, and those having lower uric acid levels. Given that these participants are likely to have fewer risk factors for renal impairment apart from their working hours, it suggests a more pronounced hazardous effect of long working hours on renal health. A previous cohort study among adult HCW indicated that increased work burden correlated with a decreased frequency of voiding daily (30). Moreover, unhealthy behaviors such as inadequate water consumption and delayed voiding are known to contribute to kidney problems (31). These issues are often more pronounced among women (32), possibly explaining why females in our study showed a significant correlation between longer working hours and lower eGFR. This finding underscores the need for better workplace practices and health awareness, especially for female HCW, to mitigate the risk of renal impairment. This finding contrasts with a previous study, which reported that working >52 hours per week was associated with incident CKD among older (age >40 years) and male subgroups (10). Our study population, with an average age 10 years younger and a predominance of female participants (compared to 75.3% males in the prior study), focused on early renal impairment rather than overt CKD. These demographic differences and the focus on early-stage renal dysfunction likely account for the discrepancies observed between our findings and those of the previous study. Consequently, there is a growing need to recognize the risk of early impaired renal function among younger people due to long working hours and night working hours. Additionally, the impact of working hours on women’s renal function warrants further attention.

A previous study demonstrated smaller within-subject biological variation in eGFR derived from the CKD-EPI and MDRD equations compared to that in measured GFR (33). In our study, we chose the CKD-EPI equation to calculate eGFR (table 2) because it exhibits less bias than the MDRD equation, especially for eGFR values exceeding 60 mL/min/1.73 m^2^ (34, 35). We also demonstrated that eGFR derived from the MDRD equation showed over-estimation and greater variability once the eGFR is >90 ml/min/1.73m^2^ (supplementary figure S1). Therefore, replacing CKD-EPI with MDRD for deriving eGFR resulted in inflated effect sizes (supplementary table S1). We found that individuals engaged in shift work had lower eGFR in model 1 (table 2), a finding consistent with previous studies (13, 36). The association between long working hours and renal impairment remained significant even after adjusting for shift work status, leading us to conclude that longer working hours are associated with lower eGFR independently of shift work status. Hyperuricemia, recognized as a well-known risk factor for kidney function (37), was strongly associated with lower eGFR across all models (table 2). Notably, the negative association of total working hours and night working hours with eGFR persisted even after adjusting for uric acid levels. Our findings regarding the age effect on kidney function aligned with previous literature, which demonstrated that eGFR typically begins to decline at a rate of approximately 0.89 ml/min/1.73 m^2^ per year after the age of 40 with a trend observed among both men and women (38). As the aging process naturally impacts kidney health, the additional strain from longer working hours and night working hours could have more implications, warranting increased vigilance and preventive strategies.

We recognize several limitations in our study. First, our sample predominantly comprised young-to- middle-aged HCW who generally enjoy better health and possess more medical knowledge than the general population. This specific demographic profile might limit the applicability of our findings to a real-world context. Although our study did not specifically assess socioeconomic status or educational background, the composition of our participants was relatively homogeneous. The Labor Standards Law does not apply to physicians in Taiwan, and records of their working hours are lacking. Therefore, we did not include physicians in our analysis. Due to the scarce number of individuals working >50 hours per week, our conclusion cannot be extrapolated to those with extremely long working hours. Several potential confounding factors were not adjusted, including physical activity, use of renal toxic drugs, family history of CKD, autoimmune diseases like lupus nephritis, and history of renal stones. To mitigate the impact of pre-existing kidney conditions on our findings, we excluded subjects with pre-enrollment CKD (eGFR <60 ml/min/1.73 m^2^) and proteinuria. Additionally, long working hours and night work may contribute to sleep deficiency (18). The duration and quality of sleep were further associated with CKD (39). However, information on sleep was lacking in our study. We observed that the study participants had a mean follow-up duration of 6.2 years, over a study period of 19 years (2002–2021). New employees join the cohort during the study period, making this a dynamic cohort, not a closed one. Notably, half of the participants were enrolled after 2009. Moreover, HCW in Taiwan exhibit a high turnover rate. We conducted further analysis on participants who resigned before the study end. The mean age at resignation was 30.5 years, while the mean age at the last follow-up for those who remained in the study was 37.8 years. Consequently, the potential for healthy worker bias in our study could be considered negligible. Lastly, we were unable to access work experience data prior to 2002, preventing us from evaluating the long-term effects of total work years on renal function.

In conclusion, HCW with longer working hours or night work are at risk of lower renal function. This highlights the need for attention towards early renal impairment, particularly among those with seemingly fewer CKD risk factors, such as younger age and female gender. It is imperative for hospitals to implement preventive programs aimed at protecting workers from developing CKD. Moreover, we recommend that labor protection policies be strengthened, advocating for the government to enforce stricter regulations on working hours and to integrate health promotion programs within these regulations. Additionally, further research is needed to determine the safe thresholds for working and night working hours to mitigate the risk of renal function impairment.

## Supplementary material

Supplementary material
