# Supplementary material for: Impaired kidney function among young healthcare workers with long working hours and night work
Source: Scand J Work Environ Health. 2024 Jun 27;50(5):380–9. doi: 10.5271/sjweh.4159 (PMC11247656; doi:10.5271/sjweh.4159)
Supplement: Supplementary material [file SJWEH-50-380-S001.pdf]

Impaired kidney function among young healthcare workers with long working hours and night work[1]

by Wan-Chin Chen, MD, Hsiao-Yu Yang MD, MS, PhD [2]

1. *Supplementary material*
2. *Correspondence to: Hsiao-Yu Yang, Institute of Environmental and Occupational Health Sciences, National Taiwan University College of Public Health, No. 17 Xuzhou Road, Zhongzheng Dist., Taipei, Taiwan. [E-mail: hyang@ntu.edu.tw]*

Supplementary Table S1. Fixed effects parameters of the generalized linear mixed model with estimated glomerular filtration rate (MDRD) as outcome (n=10677).

	β (95% CI)		
	Model 1	Model 2	Model 3
Total working hours[a]	**-3.69 (-4.51 – -2.87)**	-	-
Night working hours[a]	-	**-1.00 (-1.48 – -0.52)**	-
Non-night working hours[a]	-	-	**-0.51 (-0.97 – -0.06)**
Age (years)	**-0.80 (-0.88 – -0.72)**	**-0.81 (-0.89 – -0.73)**	**-0.78 (-0.85 – -0.70)**
Follow-up time (years)	**-1.16 (-1.25 – -1.07)**	**-1.11 (-1.20 – -1.03)**	**-1.10 (-1.19 – -1.01)**
Male sex	**-4.81 (-6.60 – -3.02)**	**-5.09 (-6.88 – -3.30)**	**-4.89 (-6.68 – -3.09)**
Hypertension	**-1.42 (-2.23 – -0.60)**	**-1.50 (-2.32 – -0.69)**	**-1.58 (-2.40 – -0.76)**
Diabetes	-0.11 (-2.21 – 2.00)	-0.06 (-2.17 – 2.04)	-0.07 (-2.17 – 2.04)
BMI (kg/m^2)	**1.16 (0.98 – 1.33)**	**1.23 (1.05 – 1.40)**	**1.21 (1.03 – 1.39)**
HDL-C (mg/dL)[b]	**2.04 (1.68 – 2.40)**	**2.20 (1.84 – 2.56)**	**2.19 (1.83 – 2.56)**
LDL-C (mg/dL)[b]	**-0.21 (-0.37 – -0.06)**	**-0.18 (-0.34 – -0.02)**	**-0.19 (-0.35 – -0.03)**
TG (mg/dL)[b]	**0.47 (0.38 – 0.56)**	**0.49 (0.40 – 0.59)**	**0.50 (0.40 – 0.59)**
Uric acid (mg/dL)	**-8.44 (-8.89 – -7.98)**	**-8.47 (-8.93 – -8.01)**	**-8.46 (-8.92 – -8.00)**
Current smoker	-2.62 (-7.30 – 2.06)	-2.48 (-7.28 – 2.31)	-2.69 (-7.50 – 2.13)
Drinking	-0.38 (-2.63 – 1.87)	-0.52 (-2.78 – 1.74)	-0.50 (-2.75 – 1.76)
Shift work	**-0.93 (-1.74 – -0.11)**	-	-

Night working hours were from 0:00 am to 8:00 am. Total working hours were considered as the sum of all working hours, encompassing both non-night and night working hours.
Model 1, 2, and 3 adopt total working hours, night working hours, and non-night working hours as main predictors, respectively. Model 1 additionally adjusted for shift work status.
[a] For every 10 hours per week
[b] For every 10 mg/dL
Numbers in bold indicated statistically significant findings ($p < 0.05$).
Abbreviations: SBP, systolic blood pressure; DBP, diastolic blood pressure; BMI, body mass index; HDL-C, high-density lipoprotein cholesterol; LDL-C, low-density lipoprotein; TG, triglycerides; eGFR, estimated Glomerular filtration rate.

Supplementary Table S2. Fixed effects parameters of the generalized linear mixed model with estimated glomerular filtration rate (different formula) as outcome (n=10677).

	β (95% CI)	
	2021 CKD-EPI	MDRD
Total working hours		-
<30/week	Reference	Reference
30 – 40/week	**-1.27 (-1.66 – -0.87)**	**-5.62 (-7.06 – -4.18)**
>40/week	**-1.23 (-1.72 – -0.74)**	**-5.30 (-6.97 – -3.62)**
Age (years)	**-0.76 (-0.79 – -0.73)**	**-0.80 (-0.88 – -0.72)**
Follow-up time (years)	**-0.87 (-0.90 – -0.83)**	**-1.12 (-1.21 – -1.03)**
Male sex	**-4.47 (-5.45 – -3.50)**	**-5.05 (-6.84 – -3.26)**
Hypertension	**-0.38 (-0.71 – -0.05)**	**-1.46 (-2.27 – -0.64)**
Diabetes	-0.08 (-0.89 – 0.73)	-0.07 (-2.17 – 2.03)
BMI (kg/m^2)	**0.34 (0.27 – 0.40)**	**1.17 (1.00 – 1.35)**
HDL-C (mg/dL)[b]	**0.28 (0.17 – 0.38)**	**2.08 (1.71 – 2.44)**
LDL-C (mg/dL)[b]	**-0.08 (-0.14 – -0.03)**	**-0.19 (-0.35 – -0.04)**
TG (mg/dL)[b]	**0.09 (0.07 – 0.12)**	**0.47 (0.38 – 0.57)**
Uric acid (mg/dL)	**-2.87 (-3.07 – -2.66)**	**-8.46 (-8.91 – -8.00)**
Current smoker	-0.96 (-3.02 – 1.10)	-2.51 (-7.23 – 2.22)
Drinking	-0.55 (-1.53 – 0.44)	-0.50 (-2.75 – 1.75)
Shift work	**-0.36 (-0.63 – -0.09)**	**-1.20 (-2.03 – -0.38)**

Night working hours were from 0:00 am to 8:00 am. Total working hours were considered as the sum of all working hours, encompassing both non-night and night working hours.
The measurement of eGFR (the response) is 2021 CKD-EPI and MDRD, respectively. Both models adopted categorical levels of total work hours as main predictors.
[a] Categorized into three levels and <30 hours per week as the reference
[b] For every 10 mg/dL
Numbers in bold indicated statistically significant findings ($p < 0.05$).
Abbreviations: SBP, systolic blood pressure; DBP, diastolic blood pressure; BMI, body mass index; HDL-C, high-density lipoprotein cholesterol; LDL-C, low-density lipoprotein; TG, triglycerides; eGFR, estimated Glomerular filtration rate.

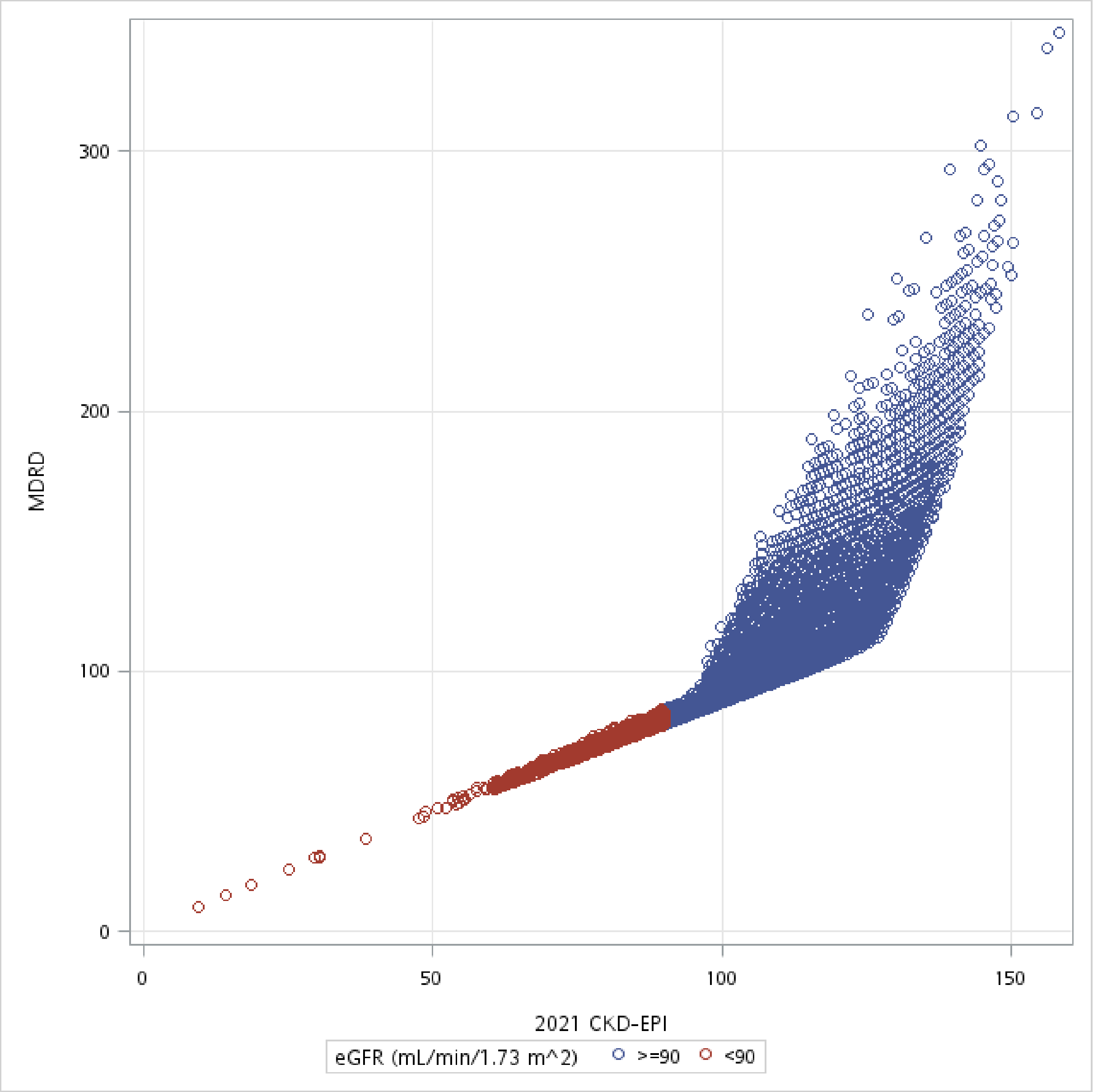

Supplementary Figure S1. A scatterplot illustrating the relationship of eGFRs derived from 2021 CKD-EPI and MDRD equations.
